# A Sustainable Near-Peer Teaching Model for Novice Anaesthetists

**DOI:** 10.7759/cureus.81334

**Published:** 2025-03-28

**Authors:** Sophie Hunter, Ee Jane Lim

**Affiliations:** 1 Anaesthetics, The Royal Free Hospital, London, GBR

**Keywords:** medical education, near peer teaching, novice anaesthetists, peer support, primary frca

## Abstract

Introduction

Anaesthetic exams are challenging and require doctors in training (residents) to study alongside performing their clinical duties. Near-peer teaching involves students who are more advanced in a subject teaching other students. This teaching model is based on the Self Determination Theory to encourage trainee self-motivation in anaesthetic training. In this study, we aimed to determine if this could be implemented within a local teaching programme.

Methods

A cohort of 16 Novice Anaesthetists (NA) participated in a protected fortnightly teaching programme lasting half a day to cover a key theme of the Primary Fellowship of the Royal College of Anaesthetists (FRCA) syllabus. ‘Bite-sized’ topics were allocated to participants who were given 45 minutes to research their topics before presenting back to their colleagues. A post-Primary FRCA resident facilitated peer-led discussions and ensured that topics were explored appropriately throughout each session. Likert scale and free text questionnaires were used to obtain feedback from those involved and other educational stakeholders and was collated pre- and six months post-implementation of the teaching programme.

Results

All 16 (100%) residents felt that the small-group teaching was conducive to their learning, increased learner confidence, understanding and ability to talk through difficult topics. Feedback obtained from College Tutors indicates that this teaching model improves teaching delivery without requiring a large faculty. Enabling senior trainees to facilitate while using their Educational Development Time (EDT) ensures sustainability. All residents who partook in the programme were more likely to carry out personal study outside of teaching and would recommend this teaching programme to incoming anaesthetists.

Conclusions

This study demonstrates a high-impact, sustainable and simple-to-implement teaching model. Its key strength lies in encouraging independent, autonomous learning to take place, with senior guidance.

## Introduction

The Primary Fellowship of the Royal College of Anaesthetists (FRCA) is a challenging exam with a wide and extensive syllabus to cover. Resident doctors (fully qualified doctors in postgraduate training or employed in non-training posts) regularly face difficulties in passing these exams [1], especially whilst working an on-call rota and maintaining a work-life balance. We looked into the feasibility of supplementing an established regional teaching programme with a local, peer-led teaching model. This was rolled out across two London teaching hospitals, both of which have an annual intake of novice anaesthetists (NA). The benefits of peer-led teaching have previously been well documented, and it is known to create a comfortable setting to develop understanding and provide a less intimidating learning environment [2].

The Primary FRCA consists of two parts: the written exam comprising Multiple Choice Questions (MCQ), followed by an Objective Structured Clinical Examination (OSCE) and Structured Oral Examination (SOE) [3]. The latter two cannot be attempted until the MCQ exam has been passed. In two London teaching hospitals, we identified a need to incorporate both local and regional teaching for the Primary FRCA into NA training to help meet the needs of resident anaesthetists and support them in their examination attempts.

The impact of the coronavirus disease 2019 (COVID-19) pandemic on UK anaesthetic training has been well documented. In a 2023 survey published in the British Journal of Anaesthesia, 76% of respondents felt that the pandemic had negatively impacted their examination experience [4], and of note, a lack of normal teaching was highlighted [4]. Teaching during normal working hours requires commitment from both the trainer and trainee, with maintenance of safe staffing levels for clinical work to continue. Near-peer teaching (NPT) is a feasible alternative to the more resource-intensive trainer or consultant-led teaching model that has been historically used in postgraduate teaching [5]. NPT involves students who are more advanced in a subject teaching other students [6]; this teaching involves a more senior anaesthetist, specifically one who has passed the Primary FRCA examinations in full, teaching NAs who are preparing for the examinations. An NPT programme was implemented based on the Self Determination Theory [7]. This was specifically chosen to enable and encourage trainee self-motivation in anaesthetic training, and we aimed to determine if this could be implemented within a local teaching programme.

This article was previously presented as a poster at the 2023 Anaesthesia Conference on May 17, 2023.

## Materials and methods

Programme structure

The programme involved two teaching sites. Teaching site one had nine participants, whilst teaching site two had seven participants. Protected teaching time was allocated to trainees, and those who were at work during teaching days were taken off afternoon clinical work and allocated a half day for each session. The sessions ran locally in the departmental seminar room and the use of a whiteboard was available if required. This programme ran for six months in total at each teaching site from November 2022 to July 2023. The model is explained in detail in Table 1.

**Table 1:** Breakdown of teaching model components

Teaching model components	Values
Total no. of participants	16 residents
Total duration of programme	Six months
Session frequency	Fortnightly
Total duration of each session	Four hours
Total facilitators required per session	One facilitator

**Figure 1:**
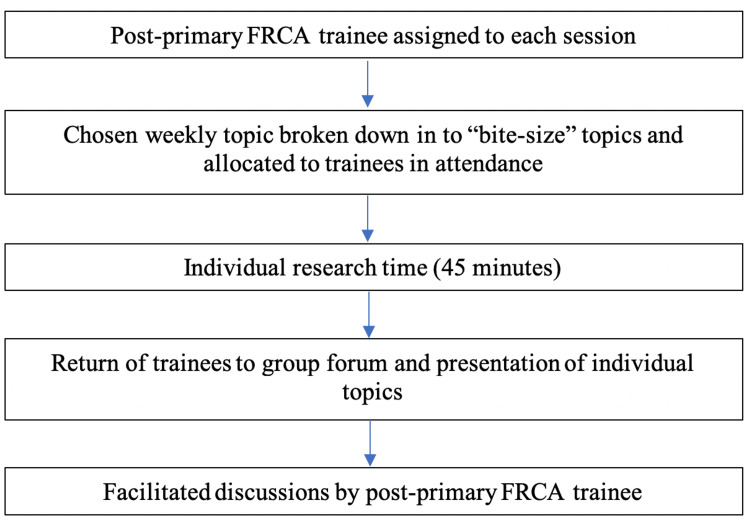
Key structure of each session FRCA: Fellowship of the Royal College of Anaesthesia

Sessions were developed based around the Primary FRCA syllabus and this was broken down into distinct topics that would be “bite-sized” in nature, with each session covering a key theme. Topics were allocated to trainees at the start of each session. They were subsequently given 45 minutes to research their allocated topic before presenting it back to their peers. Research was conducted using textbooks available in the department or internet searches on personal devices. A post-Primary FRCA resident was present at each session and oversaw both the allocation of topics and key points presented (Figure 1). They ensured that topics were explored appropriately during sessions and were able to facilitate relevant peer-led discussions. When trainees had questions or needed clarification on certain aspects of the syllabus, the post-Primary FRCA resident was able to guide and teach. Facilitating trainees were also permitted to use their Educational Development Time (EDT) to lead these sessions and gain teaching experience.

Teacher recruitment was carried out via departmental broadcast communication, email and at weekly departmental meetings. The programme was presented on multiple occasions to ensure that all eligible trainees were made aware, accounting for variations in on-call rota patterns that may have hindered attendance at weekly departmental meetings. Any trainees who did not want to partake in the programme were allowed to decline.

Feedback was collated pre- and six months post-implementation of the teaching programme using Likert scale questionnaires. Both qualitative and quantitative data from either free-text options or the five-point scale response score were collected. The pre-course questionnaire identified how well received the teaching programme would be and identified what trainees would hope to gain from the programme (Appendix A). The post-course questionnaire was disseminated six months after the initial programme had been implemented (Appendix A). 

Individual session feedback was collected after each session had been delivered and provided facilitators with feedback on their teaching. This was collected to present session facilitators with written feedback for their portfolio development and evidencing teaching experience. Feedback from the College Tutors, who were the appointed educational leaders in the department overseeing anaesthetic trainees, was obtained at the end of the initial six-month period (Appendix B). This established how well the model worked in the department and the wider effects it would have on departmental staffing.

## Results

Sixteen (100%) residents who were offered the programme partook, and all completed the pre-course survey across both teaching sites. Of them, 10 (63%) residents responded that they received no formal teaching before the programme, and five (31%) residents stated that they received formal teaching once a month. All 16 (100%) residents wanted to attend the teaching programme irrespective of if they were planning to sit the exam within the next year. Six (38%) residents planned to sit the exam within the next year. When asked if residents felt that the standard regional teaching days were sufficient for their preparations for the Primary FRCA exam, 13 (81%) strongly disagreed. Thirteen (81%) residents also felt that weekly teaching would be the desired frequency of teaching sessions. When considering the impact of teaching time on clinical experience, 16 (100%) residents felt this would not be detrimental both pre- and post-implementation of the teaching programme. Six (38%) residents strongly disagreed when asked if they felt confident to start revising for the Primary FRCA, with 14 (88%) disagreeing overall.

Preliminary feedback in the pre-course questionnaire was collected to identify how well received the teaching programme would be and what trainees would hope to gain from the programme. Freetext answers are listed in Table 2.

**Table 2:** Key responses to the question “What might you find helpful and want to see as part of your Novice teaching sessions” FRCA: Fellowship of the Royal College of Anaesthesia

Responses to commencing the teaching programme	Session requests
“Our current formal teaching has been less than monthly - I would greatly appreciate additional teaching if this can be organised for us”	“Topics relevant to the Primary and how to apply them to clinical practice”
“Please teach us Primary topics”	“It would be great to have some basic principles covered”
“Primary teaching would be great and very helpful to direct our own personal study”	“Key concepts for the Primary FRCA, specifically including physics”
“I would be very keen for small group teaching”	“Challenging concepts in the Primary syllabus”
“Regular teaching would be very helpful for my training and preparing for the Primary FRCA”	“Recommendations for appropriate courses”

**Figure 2:**
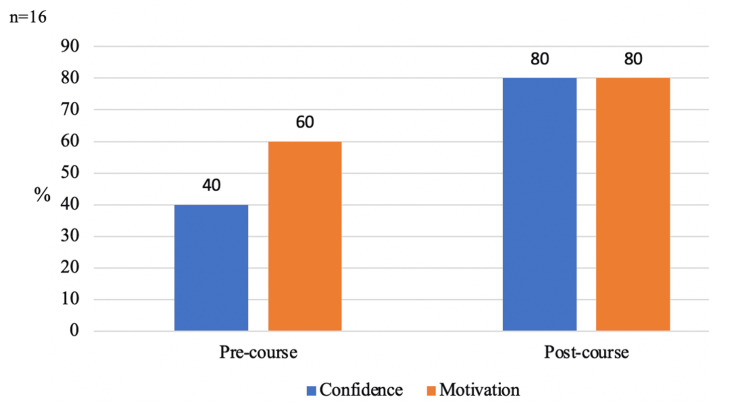
Outcomes pre- and post-implementation of the teaching programme Both confidence and motivation to study for the Primary FRCA increased six months after course implementation FRCA: Fellowship of the Royal College of Anaesthesia

Six months after the implementation of the teaching programme, the same questionnaire was circulated to the 16 programme participants. Fifteen (94%) residents responded to the post-course survey. Figure 2 summarises the improvements seen in both confidence and motivation for preparing for the Primary FRCA.

Freetext answers collating feedback from the post-course questionnaire on individual experiences of the teaching programme can be seen in Table 3.

**Table 3:** Key feedback obtained in the post-course survey in response to the instruction “Please add any other comments about your experience of the teaching programme” COVID-19: coronavirus disease 2019; FRCA: Fellowship of the Royal College of Anaesthesia

Comments about the programme	Suggestions for future development
“The teaching has been fantastic”	“It would have been good if the programme had been started earlier in our training year”
“The programme has helped me understand key topics likely to be tested in the Primary FRCA and the structure was effective”	“More frequent teaching would be of benefit”
“This teaching was amazing, not didactic, very relevant and an important social opportunity, which due to COVID-19 has been sorely lacking from training so far”	“I was disappointed that the programme started so late in the year as it would have been great to have it earlier”
“Thank you for this great teaching. It has really helped me in preparation for the Primary FRCA and has been a valuable opportunity to get together with fellow Novices to discuss topics, which otherwise we would not have been able to do”	“Having some consultant-led sessions may help improve sustainability of the course around resident rotation times”
“This teaching programme is the best teaching I’ve ever attended. It’s so well organised and uses such strong educational principles to maximise engagement and retention”	“This programme started two-thirds of the way through the year, it would have been better if it started much earlier”
“Teaching at this hospital has otherwise been poor or non-existent, and I feel that without this teaching, I would not even know how to revise. I’m really grateful”	“Continue with the peer-led group- it has been great to have the support of colleagues at similar levels and the chance to seek advice from senior residents too”

In the pre and post-course questionnaire form, we assessed how well received the small group learning environment was. The results of this can be seen in Table 4. Six (67%) residents at teaching site one and six (86%) residents at teaching site two strongly agreed that a small-group teaching environment would be conducive to their learning in the pre-course questionnaire. In the post-course questionnaire, this rose to eight (89%) residents at teaching site one and seven (100%) at teaching site two (Table 4).

**Table 4:** Comparison between the responses of the two teaching sites to the question “I strongly agree that small group tutorials are a good environment for me to ask questions, check my understanding and learn interactively”

	Teaching site one (nine participants)	Teaching site two (seven participants)
Pre-course	6 (67%)	6 (86%)
Post-course	8 (89%)	7 (100%)

Feedback from the College Tutors was obtained at the end of the initial six-month period. Freetext answers can be seen in Table 5.

**Table 5:** Key feedback obtained from College Tutors six months after the implementation of the teaching programme FRCA: Fellowship of the Royal College of Anaesthesia; NA: Novice Anaesthetist

Questions asked	College Tutor responses
How, as trainers, have you found the structure of this mode of teaching?	“This model works brilliantly for NAs. Because it requires active participation, ability to digest and explain concepts, it helps individuals develop skills necessary for the oral FRCA, whilst building trust and rapport between the peer group”
How sustainable do you feel this model is for the department?	“Sustainable teaching programme in a busy clinical setting. The topics covered complement the in-situ teaching in theatre. Because you approached the College Tutors and rota team, we were able to find an appropriate time in the week for protected teaching, and alternate weeks means minimal negative impact on clinical training”
How likely are you to continue this programme with the next group of residents?	“Given the success and positive feedback, we intend to continue the teaching programme for subsequent groups of core trainees”
What considerations would you recommend for other departments interested in adopting this style of local teaching?	“A high impact education project that has undoubtedly enhanced the training and experience of our current NA cohort. We now feel we have the ability to continue to implement it for all future cohorts”

## Discussion

We identified a significant gap in teaching provision for NAs and a lack of support in preparation for the demands of the Primary FRCA in our local hospitals. This has recently been noted on a wider national scale following the COVID-19 pandemic [4]. We have demonstrated the request from resident anaesthetists to have a supported teaching programme to supplement regional teaching days. We have shown how this model can be implemented in two busy London teaching hospitals and woven into the clinical work schedule with minimal impact on clinical experience. Our results are overall very positive regarding the implementation of the programme, increasing learner confidence and their ability to discuss difficult concepts, in a safe learning environment. The sustainability of the model we present here is key to its success. As previously acknowledged, peer-led teaching is a feasible alternative to the more resource-intensive trainer or consultant-led teaching models [5]. This model ultimately encourages independent learning to take place, with senior guidance, which improves programme longevity [8] and is already widely used across many UK medical schools [9].

Maintaining a strong working relationship with the local faculty, in this case, the College Tutors, was key to successfully implementing the programme. The importance of this relationship has been noted previously in similar medical school teaching models [8]. It was key to ensuring adequate EDT could be secured regularly for NAs and teachers. We worked with them to create a model that would be easy to pass on to incoming residents at times of rotation. Moreover, this model is particularly geared towards implementation in an anaesthetic department. The nature of UK anaesthetic training often features one-to-one teaching in theatre: a consultant and a trainee [10]. Theatre lists can often feasibly run solely with a consultant. This lends itself to enabling trainees to be “released” from lists to attend teaching fortnightly without disrupting clinical work. We specifically opted to run the programme in the afternoon so that pre-assessments and general preparations for the overall list would be completed in the morning, relieving pressure on the consultant running each list solo in the afternoon. Again, this contributed to the programme being positively received by the consultant body as well as increasing its longevity.

In recent years, an increase in online teaching and disruptions to in-person teaching sessions contributed to increased feelings of social isolation amongst medical students and doctors worldwide [11]. One of the hidden benefits of this programme is the peer support for similar stage trainees to gather, learn and gain support from each other, the benefit of which has been well documented [12]. The positive impacts on psychological well-being amongst residents were noted in the feedback obtained. The presence of senior resident anaesthetists allowed for guidance as well as advice to be given, from those who had previously passed exams.

This model relied on several factors: dedicated trainees who wanted to partake in an NPT experience, committed facilitating residents and support from the College Tutors. From the outset, it was intended to produce a teaching model that was sustainable and replicable across many hospital sites. Due to the rotational nature of anaesthetic training in the UK, rotations to different hospitals can occur as frequently as every three months. The detrimental impact this can have on trainee wellbeing and teaching has been previously documented [13]. We wanted to ensure that this model was accommodating to trainees present in departments for short or longer periods of time. We wanted to design a model that could be accommodated by the department and ensure that regular EDT could be allocated to trainees to allow for the continuity and sustainability of the teaching programme. This required support from the consultant body and forward planning of list allocations, allowing for trainees to reliably be “released” from afternoon clinical lists on set days. Close work with the College Tutors and their support with creating the model was key. This has also proven crucial in the continuation and longevity of this teaching programme, which continues in both teaching sites to date.

Study limitations

The small sample size of our study represents only a proportion of the national anaesthetic trainee population. Future steps in this programme would include development and implementation at a national level to assess response and to ensure it can be accommodated by multiple UK anaesthetic departments. No formal teacher training was provided to post-Primary residents who facilitated sessions and no comparison to other local teaching models was conducted. In future, teacher training could be provided to enhance the experience both for NAs and teachers, besides standardising the teaching and supervision provided when expanding this to more centres.

## Conclusions

We discussed a high-impact, sustainable and simple-to-implement NPT model. This programme has increased learner confidence, understanding and ability to talk through difficult topics. Its strength lies in encouraging independent, autonomous learning to take place, with senior guidance. Following this programme, all NAs who partook in the programme were more likely to carry out personal study outside of teaching and would recommend this teaching programme to incoming NAs. Further research is needed to investigate the successful implementation of this teaching programme on a wider scale.

## Appendices

Appendix A: pre-course and post-course questionnaire

Please answer the following questions

1) How much formal teaching do you receive at the moment? Please select from the following options

- None

- Once a month

- More than once a month

- Once a week

- More than once a week

2) I would like FRCA-syllabus teaching regardless of whether I intend to sit the Primary exam in the next year. Please select from the following options

- True - I intend to sit the Primary this year AND would like FRCA-syllabus teaching

- True - I do NOT intend to sit the Primary this year AND would like FRCA-syllabus teaching

- False - I intend to sit the Primary this year AND would NOT like FRCA-syllabus teaching

- False - I do NOT intend to sit the Primary this year AND would NOT like FRCA-syllabus teaching

Please rate the following questions on the Likert scale: 0 = strongly disagree, 5 = strongly agree

3) I feel that ONLY having regional teaching for core trainees is sufficient for my training 

4) I would like teaching to be organised on a weekly basis

5) I feel that weekly half-day teaching for core trainees will NEGATIVELY affect my clinical experience

6) I feel confident that I am able to revise for the Primary FRCA

7) I am motivated to prepare for the Primary FRCA

8) I am confident in talking about and explaining basic anaesthetic principles relevant to the Primary FRCA

9) Small group tutorials are a good environment for me to ask questions, check my understanding and learn interactively

10) Please add any other comments about what you might find helpful and want to see as part of your CT1 teaching sessions

Appendix B: College Tutor feedback request form

1) How, as trainers, have you found the structure of this mode of teaching?

2) How sustainable do you feel this model is for the department?

3) How likely are you to continue this programme with the next group of residents?

4) What considerations would you recommend for other departments interested in adopting this style of local teaching?
